# Cost-effectiveness analysis of AI-assisted chest X-ray interpretation tools for TB screening: a rapid HTA

**DOI:** 10.3389/fdgth.2025.1629127

**Published:** 2025-12-05

**Authors:** Devang Raval, Dhaval Parmar, Somen Saha, Raju Sarkar, Medha Wadhwa, Apurvakumar Pandya, Harsh Shah, Kavitha Rajsekar

**Affiliations:** 1 Indian Institute of Public Health Gandhinagar (IIPHG), Gandhinagar, India; 2 Department of Health Research, New Delhi, India

**Keywords:** AI, tuberculosis screening, HTA, cost-effectiveness, chest X-ray

## Abstract

**Background:**

Early diagnosis remains one of the major barriers to treating and managing tuberculosis (TB). Artificial intelligence (AI) has increased in importance worldwide and has been employed in the context of tuberculosis screening. This study assessed whether newer AI-assisted technologies provide cost-effective benefits for the diagnosis of pulmonary tuberculosis in resource-limited settings.

**Methods:**

This retrospective study analyzed secondary data from patients who underwent tuberculosis screening using chest x-rays interpreted by AI-assisted software (qXR and Genki) in 2023. Pooled diagnostic accuracy was calculated using the secondary literature, and cost-effectiveness was assessed by comparing the newer technology with the conventional method, i.e., manual interpretation by radiologists using digital X-rays. The cost-effectiveness analysis was undertaken using the Health Technology Assessment in India (HTAIn) guidelines. The incremental cost per additional interpreted case was the outcome.

**Findings:**

The incremental cost-effectiveness ratio (ICER) for qXR was Indian rupee (INR) −9,865 [−120 United States dollars (USD)] per interpreted case, which showed that the method was cost-saving, while for Genki, the corresponding value was INR 11,287 (137 USD), which showed that the method was cost-effective. Both ICER values were below India's per capita GDP for 2022. A threshold analysis showed that healthcare systems could spend a maximum of INR 35 (USD 0.43) and INR 410 (USD 5) for Genki and qXR, respectively, to interpret one presumptive TB case.

**Interpretation:**

AI-assisted tools, such as qXR and Genki, improve TB diagnosis with high sensitivity, specificity, and cost-effectiveness, offering a valuable alternative to traditional radiologist interpretation. Thus, they are particularly beneficial in resource-limited settings such as in India, and can enhance TB detection and patient outcomes in high-volume public healthcare institutions.

**Funding:**

The Department of Health and Research, Ministry of Health and Family Welfare, Government of India

## Introduction

Tuberculosis (TB) remains a pervasive global health challenge, claiming millions of lives and posing a substantial burden, particularly in developing countries, where it is a leading cause of mortality. In 2023, 87% of all TB cases worldwide were concentrated in 30 high-burden nations, with India accounting for 26% of cases (1).

India established an aggressive “end TB” strategy by 2025 through its National Tuberculosis Elimination Program (NTEP) in response to this national health emergency (2). Over the past decades, the country has implemented various initiatives to enhance universal access to tuberculosis care, including mandatory case notifications, real-time information management systems, rapid molecular diagnostics, and standardized treatment guidelines, which have undoubtedly accelerated early diagnosis and treatment compliance (3).

Tuberculosis diagnosis remains significantly reliant on methods such as direct sputum smear microscopy, solid culture, and chest radiography, all of which require expert interpretation. Among these, chest x-rays stand out as the only non-sputum-based diagnostic tool, but the scarcity of experts in India poses a considerable challenge (4, 5).

Artificial intelligence (AI)-assisted chest x-ray (CXR) interpretation could be a breakthrough in TB screening, particularly in developing countries such as India. AI algorithms can identify subtle patterns and abnormalities in CXRs, which, along with quantitative measurements of TB-related lesions, can aid in monitoring disease progression and evaluating treatment efficacy (6). AI-assisted solutions have the potential to revolutionize TB detection using radiography, contributing to improved patient outcomes and global public health efforts (7, 8).

Several studies have investigated the costs associated with tuberculosis treatment, revealing a range of patient expenses, including direct and indirect costs (3, 9–13). The National TB Prevalence Survey Report calculated the overall median costs associated with tuberculosis diagnosis and treatment and the financial burdens faced by affected families (14). However, evidence on the cost-effectiveness of AI-assisted CXR interpretation tools is limited. This study was conducted to assess the cost-effectiveness of AI-assisted CXR interpretation compared with interpretation of CXRs by a radiologist in the screening of TB.

Although some studies have evaluated the diagnostic accuracy or operational feasibility of AI-based CXR interpretation, few have examined its economic value in the Indian public health setting. To the best of our knowledge, this is the first rapid health technology appraisal of the cost-effectiveness of two AI-assisted interpretation methods (qXR and Genki) versus interpretation by a radiologist in TB screening, conducted within the NTEP paradigm and based on actual program data.

## Methods

### Study design and framework

This rapid health technology assessment used available retrospective data from previous years from public health facilities that utilized the selected technologies. The study’s framework followed the PICO (Population, Intervention, Comparator, Outcome) format, as detailed in Table 1.

**Table 1 Framework of the study using the:** PICO method.

Component	Description
Population	Patients screened for potential TB-related chest pathology in the previous year.
Intervention	AI-assisted chest X-ray interpretation tools: qXR and Genki
Comparator	Digital CXR annotation by a radiologist
Outcome	1. Diagnostic accuracy of the AI-assisted CXR interpretation tools compared to that of conventional CXR interpretation. 2. ICER: cost per interpreted case

### Study perspective

This study adopted a provider perspective to assess the cost-effectiveness of different chest X-ray interpretation modalities.

### AI-assisted methods

Two AI-assisted chest x-ray interpretation methods were assessed: qXR and Genki. The Central Drugs Standard Control Organization (CDSCO) of India and the United States Food and Drug Administration (FDA) approved these technologies. Both technologies utilize AI with deep learning methods to interpret CXRs. qXR offers online solutions, and version 2 was one of three AI-assisted CXR interpretation software programs recommended by the World Health Organization (WHO) for TB screening and triage (15). Another software program, Genki, provides both offline and online triaging of chest x-rays and includes a comprehensive patient communication loop as part of its end-to-end solution.

### Data sources and collection

The study sample consisted of the reported records from 93,486 patients who had undergone chest x-rays for the screening of tuberculosis using Genki at selected public health facilities in Chennai, along with 6,281 patients who had undergone chest x-rays for the screening of tuberculosis using qXR at selected public health facilities in Maharashtra in 2023. Those in this cohort were aged 15 years and older. This cohort was compared with the records of 2,731 patients who were screened for TB via conventional digital X-ray interpretation by radiologists at the same facilities before the introduction of the AI-assisted methods. The raw data with defined variables was accessed and analyzed in close coordination with the respective state's TB officials. Information on the cost of the technologies was collected from the manufacturers and collated with the information provided by the secondary literature.

### Estimation of diagnostic accuracy

We reviewed published studies on the diagnostic effectiveness of qXR and Genki to assess the diagnostic accuracy of these AI-assisted interpretation tools. The true positive (TP), true negative (TN), false positive (FP), and false negative (FN) rates were calculated using published data from the literature to determine pooled sensitivity and specificity. In this context, TP=TB case correctly identified as TB, FP=non-TB case incorrectly identified as TB, FN=TB case incorrectly identified as non-TB, and TN=non-TB case correctly identified as non-TB.

The formulas used for pooling accounted for study weights, particularly sample sizes. These pooled measures were then applied to the primary data collected from the user department to calculate the diagnostic accuracy of the AI models compared to conventional interpretation. The outcome parameters were evaluated based on confirmed microbiological tests and radiologist/expert confirmation.

We calculated the overall accuracy of the reported area under the curve (AUC) values from the primary studies, which suggested that the cumulative AUC value was 0.820 (*p* < 0.05). This was statistically significant, indicating that the overall accuracy of the AI-assisted tools was adequate compared to the standard method.

**Figure 1:**
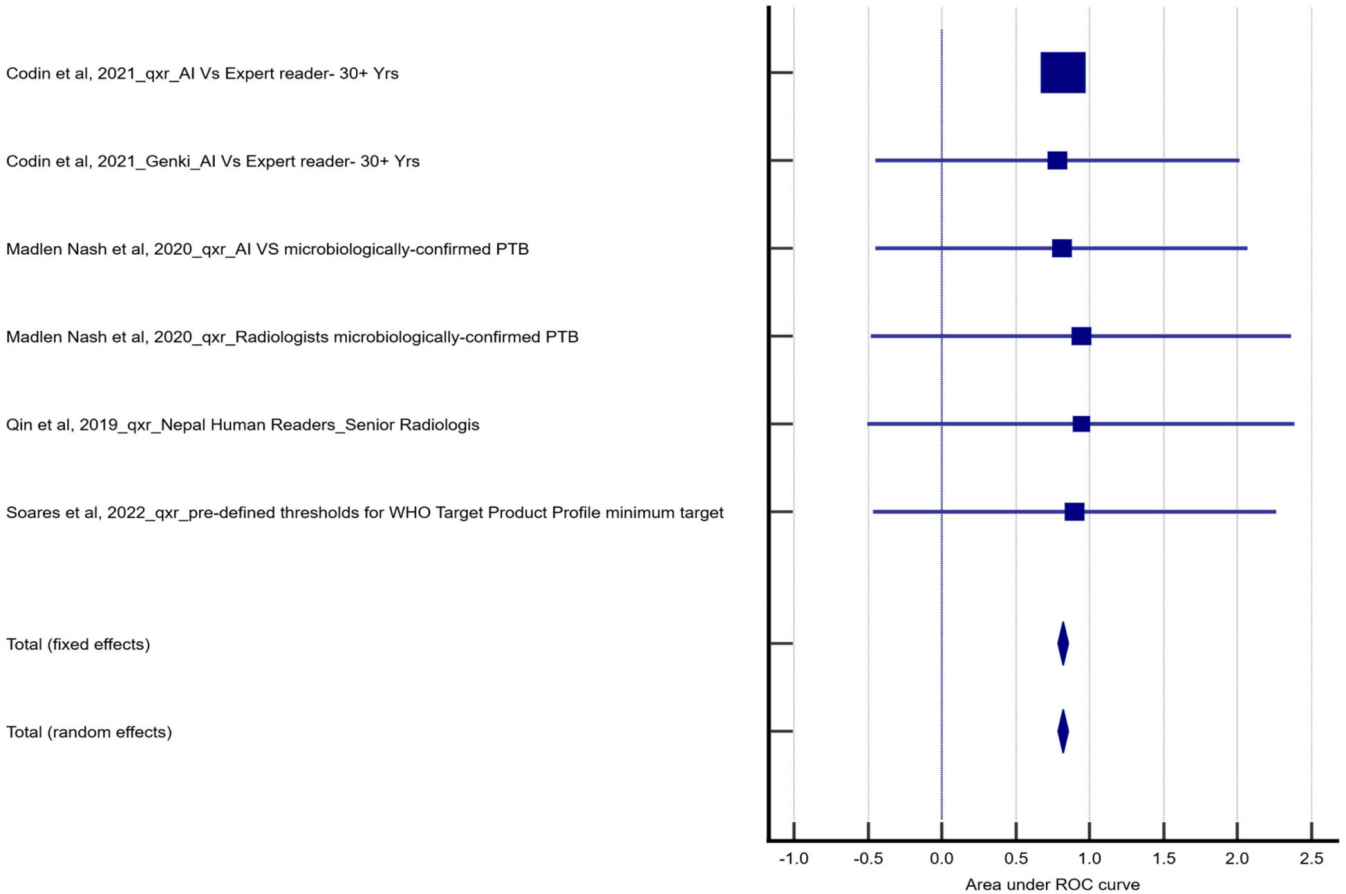
Forest plot based on receiver operating characteristic (ROC) analysis.

The pooled result revealed *I*^2^ = 0.00%; hence, there was no heterogeneity among the studies included, and this could be explained by the fact that the study designs, populations, and reference standards were similar. The forest plot in Figure 1 shows the individual weight of the studies, 95% confidence intervals, and the pooled estimate. This result suggests that, on average, the AI-assisted methods in the included studies performed well in diagnosing the condition. As shown in Table 2, the pooled sensitivity and specificity for qXR were 90.22% and 68.21%, respectively. Similarly, Genki's pooled sensitivity and specificity were estimated to be 90.41% and 66.38%. The comparator between the two AI tools yielded pooled sensitivities and specificities of 88.72% and 49.61% respectively.

**Table 2:** Diagnostic accuracy of AI-assisted tools compared to radiologist interpretation.

Authors	Sample size	Sensitivity (%)	Specificity (%)	TP^b^	FP^b^	FN^b^	TN^b^
qXR							
Codlin et al. (15)	1,032	95.5	48.7	127	461	6	438
Faiz khan et al. (16)	2,198	93	75	1,901	38	143	115
Madlen Nash et al. (17)	929	71	80	468	54	191	216
Qin et al. (18)	1,196	96	48	1,102	25	46	23
Qin et al. (7)	23,954	90.2	74.3	19,489	603	2,117	1,744
Pooled values	29,309	90.22	68.21	23,088	1,182	2,504	2,536
Genki							
Codlin et al. (15)	1,032	82	65.9	109	307	24	592
Independent evaluation by TCELS 2023^a^(19)	300	94.5	95.61	268	1	16	16
Pooled values	1,332	90.41	66.38	377	308	40	608
Interpretation by a radiologist							
Codlin et al. (15)	1,032	95.5	42.2	127	520	6	379
Codlin et al. (15)	1,032	82	57.1	109	386	24	513
Pooled values	2,064	88.72	49.61	236	906	30	892

a The Thailand Center of Excellence for Life Sciences.

b Calculated values.

The radiologist interpretation arm includes two distinct datasets reported in Codlin et al. (2021) that were analyzed separately as they represent different sub-cohorts from the same study.

### Cost analysis

The cost data for AI-assisted x-ray interpretation and conventional digital CXR encompassed capital and implementation/operational costs with a structured proforma. Direct development and setup expenditures were included in the capital costs. These costs were further broken down into infrastructure, equipment, and information technology (IT) system expenses. Operational costs comprised fixed and recurring payments, maintenance, shared manpower, training, and consumables. The costs were adjusted as per 2022–2023 prices to constant values, annualized according to their useful lifespan (maximum of 5 years), and discounted at an annual rate of 3% (20, 21). The costs were converted using 1 USD=INR 82, at the 2023 average price. The cost per interpreted case was considered the key outcome.

### Economic modeling

**Figure 2:**
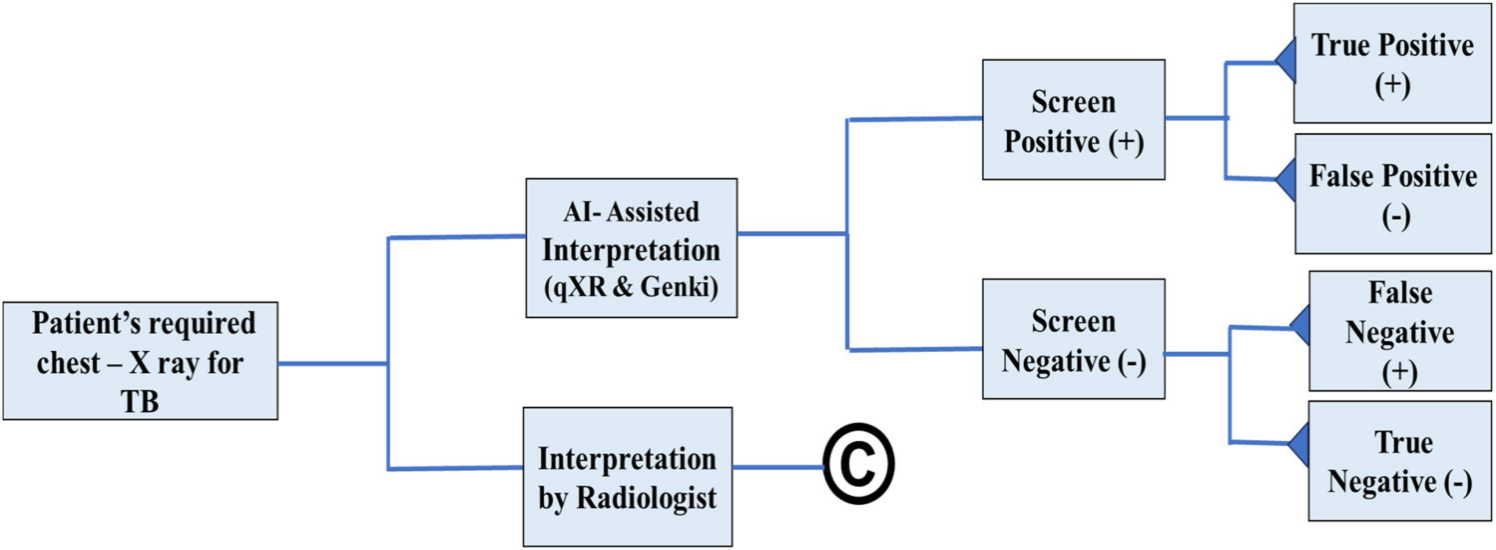
Decision tree model for AI-assisted chest X-ray interpretation.

A decision tree was used as the analytical model and parameterized using a Microsoft Excel spreadsheet to estimate the change in outcome and cost due to the implementation (deployment) of AI solutions compared to interpretation by a radiologist from a health system perspective (Figure 2). The model illustrated the various approaches, with branches representing the potential outcomes of the test, including TP, TN, FP, and FN rates. The model's outcomes were the number of interpreted cases and the total costs associated with each approach.

### Cost-effectiveness analysis

The model illustrated the potential outcomes and associated costs from a health system perspective. The analysis aimed at efficient resource allocation and cost-effectiveness. This was determined by the incremental cost-effectiveness ratio (ICER), with the thresholds set to India's GDP.

### Sensitivity and threshold analyses

A one-way sensitivity analysis was used to determine uncertainty in the model parameters using lower and upper limits with 25% or the 95% confidence interval values of the model inputs. The results were represented in tornado diagrams, and a cost-effectiveness plane was used to visualize the cost-effectiveness outcomes.

A willingness-to-pay threshold was determined to estimate the cost range at which the purchase of the AI solution would be cost-effective. ICER values were calculated by systematically increasing the cost, and the point at which these ICER values became cost-ineffective was identified. The one-time GDP per capita for the year 2022 was used as the cost-effectiveness threshold (CET), as suggested by the Indian reference case for conducting economic evaluations in health technology assessments (22).

## Results

### Cost analysis

Table 3 provides a general overview of the costs associated with providing radiology services using AI-assisted or radiologist interpretations. The cost per case for interpretation by expert radiologists was INR 100 (USD 1.2), while the AI-based technologies qXR and Genki incurred costs of INR 30 (USD 0.36) and INR 22 (USD 0.26), respectively. These results demonstrate a cost advantage for the AI-assisted screening methods. The reported cost of qXR was for the online solution only, and that for Genki covers both offline and online costs. Detailed cost breakdowns for interpretation by a radiologist, qXR, and Genki are provided in Tables 4–6, respectively.

**Table 3:** Calculation of cost per case interpreted.

Description	Cost of interpretation by a radiologist	qXR cost	Genki cost
Total cost	INR103 (USD 1.25)	INR 31 (USD 0.37)	INR 23 (USD 0.28)
After a 3% discount	INR 100 (USD 1**.**21)	INR 30 (USD 0.36)	INR 22 (USD 0.26)
Cost per interpreted case	INR 100 (USD 1.21)	INR 30 (USD 0.36)	INR 22 (USD 0.26)

**Table 4:** Detailed cost breakdown for radiologist interpretation.

Description	Cost (INR)	Remarks
Radiologist	236,400	Comparator costing adopted from a previous study conducted by the Department of Health Research (DHR), Health Technology Assessment in India (HTAIn), and Indian Institute of Public Health Gandhinagar (IIPHG). The calculation is based on a salary of INR 197,000, with 10% allocated to TB screening; this assumes approximately 55 minutes of reading time per day (calculated as 11 X-rays × 5 minutes) within a standard 6–8 hour duty shift
Data entry operator	37,554	12,518: 25% for CXR
X-ray printed	6,860	Data from two subdistrict hospitals in Maharashtra
Total cost	280,814	
Number of patients	2,731	In 2021–2022
Cost per patient	103	
Cost of interpretation per case after a 3% discount	100	A 3% discount was applied as per the HTAIn user guidelines/manual

**Table 5:** Detailed cost breakdown for AI-assisted interpretation using qXR.

Sr. no.	Description	Product	Cost (INR)	Remarks
1	Usage-based scan cost (per scan)	qXR	25	The cost of the software license includes the AI processing of chest X-rays, including all operational and capital technology costs. These include human resources, user training, deployment and integration, dedicated client support, lifecycle management, and maintenance of the software
2	Internet connection cost (shared)		1	Cost provided by the local service provider at the facility
3	X-ray printing cost		5	Standard printing cost from the facility
	Total cost^a^		31	
	Interpretation cost per case, after a 3% discount		30	A 3% discount was applied as per the HTAIn user guidelines/manual

a These costs apply to the online mode of the device. For an on-premises deployment in offline mode, a cost of INR 3 lakh (tax excluded) is applicable. The manufacturers were unable to provide a detailed breakdown due to the proprietary nature of the tool and because none of the X-ray units in India were deployed with the offline AI solution.

**Table 6:** Detailed cost breakdown for AI-assisted interpretation using Genki.

Cost breakdown of the Genki—DeepTek solution				
Sr. no.	Description	Product	Cost (INR)	Remarks
1.	Cost of the solution per unit	Genki software license	1,57,608	An offline/edge AI-based chest X-ray triaging solution that connects with any computed radiography (CR) or digital radiography (DR) machine. It can also process photos of analog scans. The system covers two pathologies (normal/abnormal and TB).
2.	Expected life of the solution	5 years	—	Unlimited scans for an unlimited period with a lifetime license. We assumed that the technology’s lifespan was a maximum of 5 years.
3.	Transportation cost of the device	Genki workstation by courier	5,000.00	A Genki workstation is delivered.
4.	Installation and training costs	In person	50,000.00	Installation and training can be done online. An in-person visit is not essential.
5.	Mode of availability in the field	Offline mode		The Genki solution works offline without the need for an internet connection.
6.	Hardware: laptop or PC	Genki workstation	16,000	The laptops or PCs needed are standard off-the-shelf products. They can be independently procured and need not be provided by us. Furthermore, the solution can be installed on the X-ray machine’s workstation (laptop/PC) if the specifications of the machine are adequate. We assumed that the technology’s lifespan was a maximum of 5 years.
7.	Server costs	RIS-VIM server per year per Genki license (i.e., per year per x-ray)	60,000	This includes features such as centralized server-based scan/data aggregation and storage, patient registration, vulnerability assessment, recording of sputum results, comprehensive analytics, and a dashboard, ensuring the efficient execution of the screening programs
		Augmento + RIS-VIM server per year per Genki license (i.e., per year per x-ray)	110,000	In addition to the above-mentioned features, this includes a zero-footprint PACS viewer, a radiologist review mechanism, smart reporting, notifications, and responsible AI, enabling tracking of the real-world performance of the AI and its biases, if any, post-deployment.
8.	Other hardware, such as Wi-Fi router, Wi-Fi receiver, and extension cord	Networking equipment	4,500	Connectivity between the X-ray machine and the Genki workstation may require additional equipment.
9.	Annual maintenance cost (AMC)	Genki AMC	50,000	AMC services are available as an optional service.
10.	Comprehensive maintenance cost (CMC)	Genki workstation	20,000	CMC services are available as an optional service.
11.	Equipment insurance		—	
12.	Total cost of one solution		2,83,108	The total applicable cost, annualized for the expected lifetime
13.	Six Tamil Nadu centers using the solution		1,698,648	
14.	Total number of patients at the facilities		93,486	(December 2022 to November 2023)
15.	Usage-based scan cost (per scan)		18	
16.	X-ray printing cost		5	Standard printing cost from the facility
	Total cost		23	
	Interpretation cost per case after a 3% discount		22	A 3% discount was applied as per the HTAIn user guidelines/manual

### Transition probabilities and model parameters

The transition probabilities were derived from published studies and calculated using standardized methods (Table 2). The details of the transition probabilities are presented in Table 7 and show the data that were considered for decision analytic modeling for the intervention and control arms. All the rates and ratios were converted into transition probabilities, and the outcomes were calculated based on various parameters assessing the diagnostic accuracy of the intervention and comparator. These encompassed TP, TN, FP, and FN rates and included the associated costs for the population under study.

**Table 7:** Calculation of transition probabilities in the decision analytic model.

Transition probability		Intervention and comparator			Remarks		
Transition from	Transition to	Transition probabilities	Value	Source	Lower bound	Upper bound	Remarks
Intervention arm: qXR							
No. of beneficiaries	No. of beneficiaries	-	6,281	Secondary	0.0000	0.0000	Data from the sites
Transition probability of positive outcome (TP + FP)	Positive	0.85	84.51	Secondary	0.8282	0.8620	Pooled sensitivity and specificity from the evidence synthesis
Transition probability of negative outcome (FN + TN)	Negative	0.15	15.49	Secondary	0.1518	0.1580	
Transition probability of screened_ TP	True positive	0.96	96.33	Secondary	0.9440	0.9825	
Transition probability of screened _ FP	False positive	0.04	3.67	Secondary	0.0360	0.0375	
Transition probability of screened _ FN	False negative	0.57	56.94	Secondary	0.5580	0.5808	
Transition probability of screened_ TN	True negative	0.43	43.06	Secondary	0.4220	0.4392	
No. of beneficiaries	No. of beneficiaries	—	6,281	Secondary	0.0000	0.0000	Data from the sites
Cost of positive (TP + FP)	Positive	1,597.91	159,791.08	Calculated	1,565.9526	1,629.8690	Calculated based on secondary data
Cost of negative (FN + TN)	Negative	292.91	29,291.02	calculated	287.0520	298.7684	
Cost of screened _TP	True positive	1,539.208	153,920.837	Calculated	1,508.4242	1,569.9925	
Cost of screened _FP	False positive	58.702	5,870.245104	Calculated	57.5284	59.8765	
Cost of screened _FN	False negative	166.775	16,677.51686	Calculated	163.4397	170.1107	
Cost of screened _TN	True negative	126.135	12,613.50102	Calculated	123.6123	128.6577	
Cost of interpretation	Cost of interpretation	0.3010	30	Primary	0.2950	0.3071	From the providers
Intervention arm: Genki							
No. of beneficiaries	No. of beneficiaries	-	93,486	Secondary	0.0000	0.0000	Data from the sites
Transition probability of positive (TP + FP)	Positive	0.85	84.96	Secondary	0.8326	0.8666	Pooled sensitivity and specificity from the evidence synthesis
Transition probability of negative (FN + TN)	Negative	0.15	15.04	Secondary	0.1474	0.1534	
Transition probability of screened_ TP	True positive	0.96	96.21	Secondary	0.9428	0.9813	
Transition probability of screened _ FP	False positive	0.04	3.79	Secondary	0.0372	0.0387	
Transition probability of Screened _ FN	False negative	0.58	57.67	Secondary	0.5651	0.5882	
Transition probability of Screened_ TN	True negative	0.42	42.33	Secondary	0.4149	0.4318	
Cost of positive (TP + FP)	Positive	17,851.65	1,785,164.91	Calculated	17,494.6162	18,208.6821	Calculated based on secondary data
Cost of negative (FN + TN)	Negative	3,159.31	315,930.75	Calculated	3,096.1213	3,222.4936	
Cost of screened _TP	True positive	17,174.253	1,717,425.335	Calculated	16,830.7683	17,517.7384	
Cost of screened _FP	False positive	677.396	67,739.57939	Calculated	663.8479	690.9437	
Cost of screened _FN	False negative	1,821.822	182,182	Calculated	1,785.3852	1,858.2580	
Cost of screened _TN	True negative	1,337.486	133,748.5856	Calculated	1,310.7361	1,364.2356	
Cost of interpretation	Cost of interpretation	0.225	22	Primary	0.2203	0.2292	From the providers
Comparator arm: radiologist interpretation							
No. of beneficiaries	No. of beneficiaries	—	2,731	Secondary	0.0000	0.0000	Data from the sites
Transition probability of positive (TP + FP)	Positive	0.84	84.40	Secondary	0.8271	0.8609	Pooled sensitivity and specificity from the evidence synthesis
Transition probability of negative (FN + TN)	Negative	0.16	15.60	Secondary	0.1529	0.1591	
Transition probability of screened TP	True positive	0.93	93.28	Secondary	0.9141	0.9514	
Transition probability of screened _ FP	False positive	0.07	6.72	Secondary	0.0659	0.0686	
Transition probability of Screened _ FN	False negative	0.64	64.08	Secondary	0.6280	0.6537	
Transition probability of screened_ TN	True negative	0.36	35.92	Secondary	0.3520	0.3663	
Cost of positive (TP + FP)	Positive	2,299.00	229,900.40	Calculated	2,253.0239	2,344.9840	Calculated based on secondary data
Cost of negative (FN + TN)	Negative	424.89	42,489.18	calculated	416.3940	433.3897	
Cost of screened _TP	True positive	2,144.41	214,441	Calculated	2,101.5190	2,187.2953	
Cost of screened _FP	False positive	154.60	15,459.7	Calculated	151.5049	157.6887	
Cost of screened _FN	False negative	272.29	27,229.0	Calculated	266.8440	277.7356	
Cost of screened _TN	True negative	152.602	15,260	Calculated	149.5500	155.6540	
Cost of interpretation	Cost of interpretation	0.997	100	Secondary	0.9775	1.0173	Calculated based on secondary data from the CXR costing study (DHR)

### Cost-effectiveness analysis

**Figure 3:**
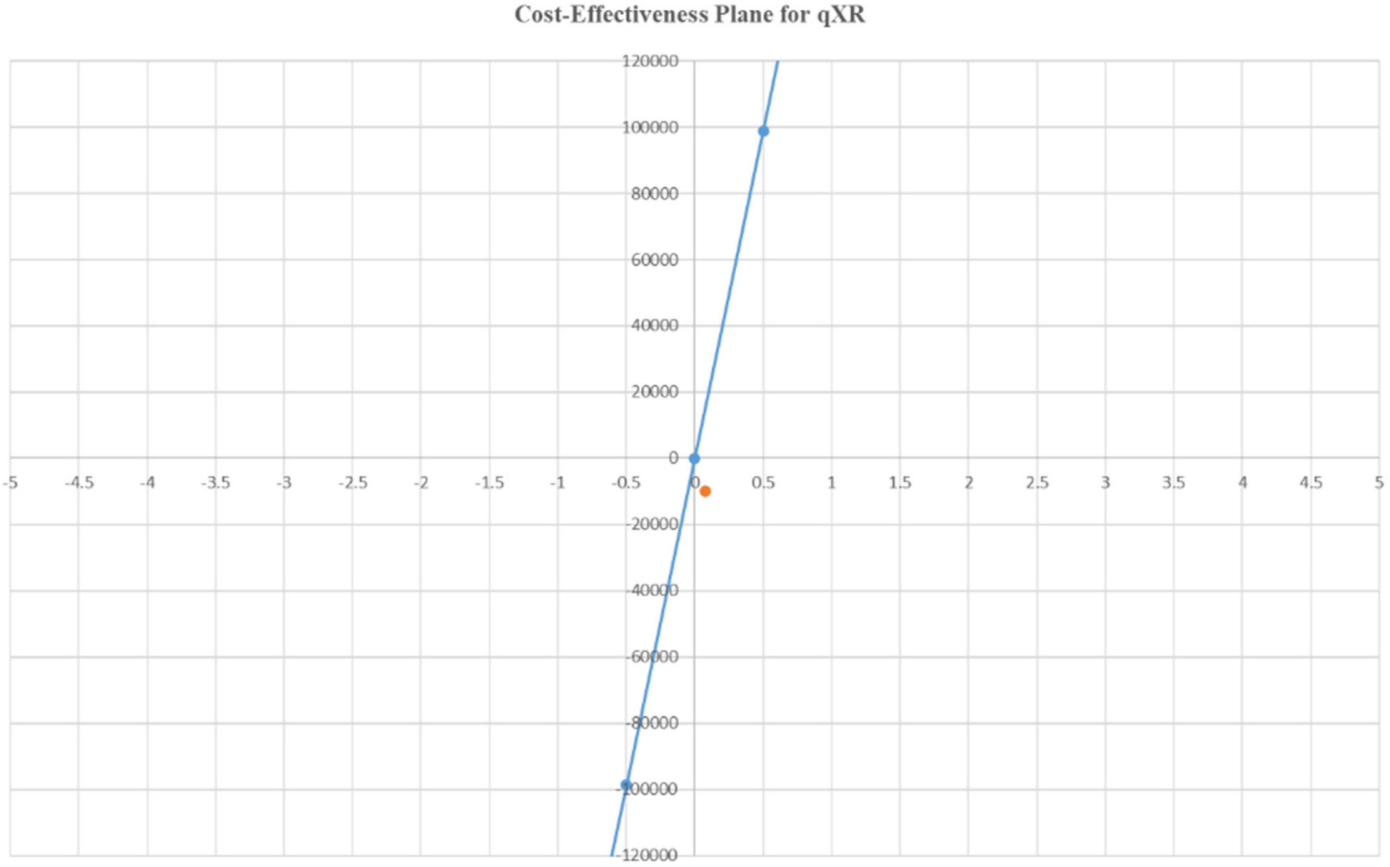
Cost-effectiveness plane for qXR.

**Figure 4:**
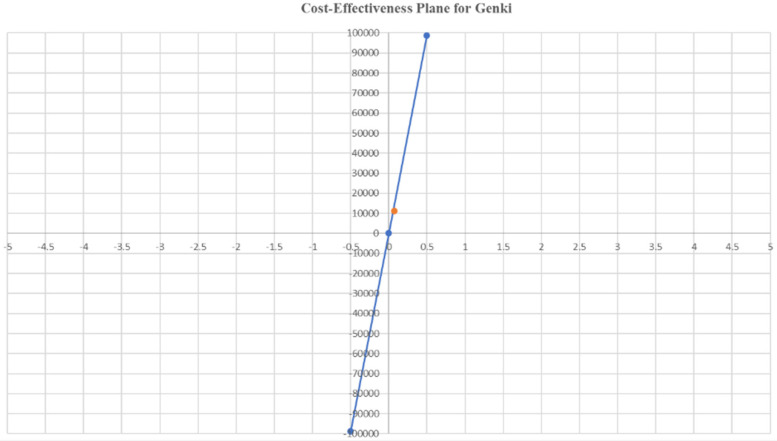
Cost-effectiveness plane for Genki.

The cost-effectiveness analyses for qXR and Genki are presented in Table 8. The ICER value per case was INR −9,864.77 (USD −120) for qXR and INR 11,286.93 (USD 137) for Genki. These values indicate that the cost of qXR and Genki is lower than that of the conventional method. The ICER values for both AI-assisted technologies were below the per capita GDP of India (2022), which was INR 197,440.48 (USD 2,408). Both interventions fell within the acceptable quadrants of the cost-effectiveness plane (Figures 3, 4), indicating favorable cost-effectiveness profiles.

**Table 8:** Incremental cost-effectiveness ratio (ICER) values.

ICER values	qXR	Genki
Difference in cost	−740.29	841.02
Detected differences in case outcomes	0.075	0.075
ICER	INR −9,864.77 (USD −120)	INR 11,286.93 (USD 137)

### Sensitivity analysis

**Figure 5:**
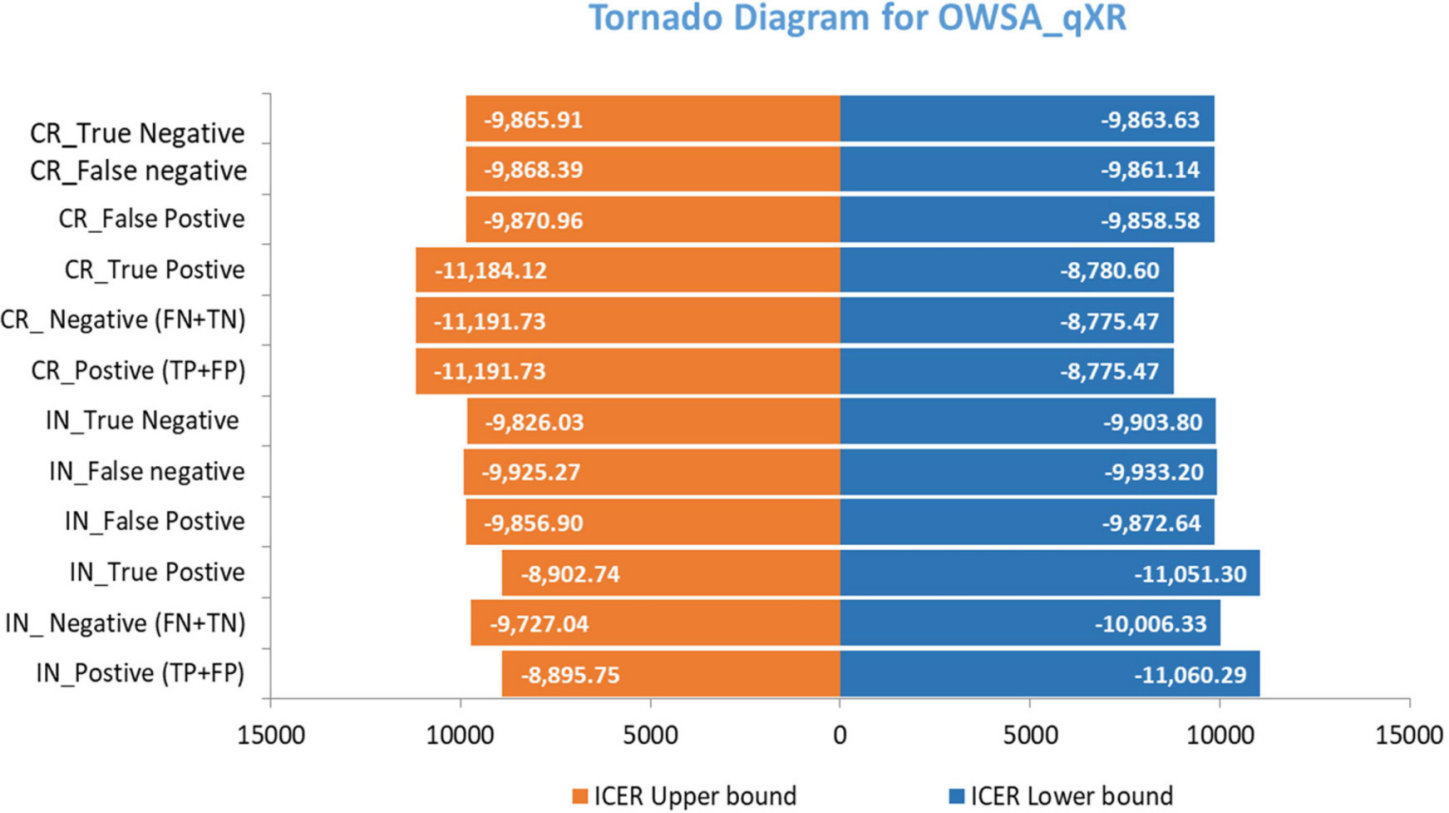
One-way sensitivity analysis for qXR.

**Figure 6:**
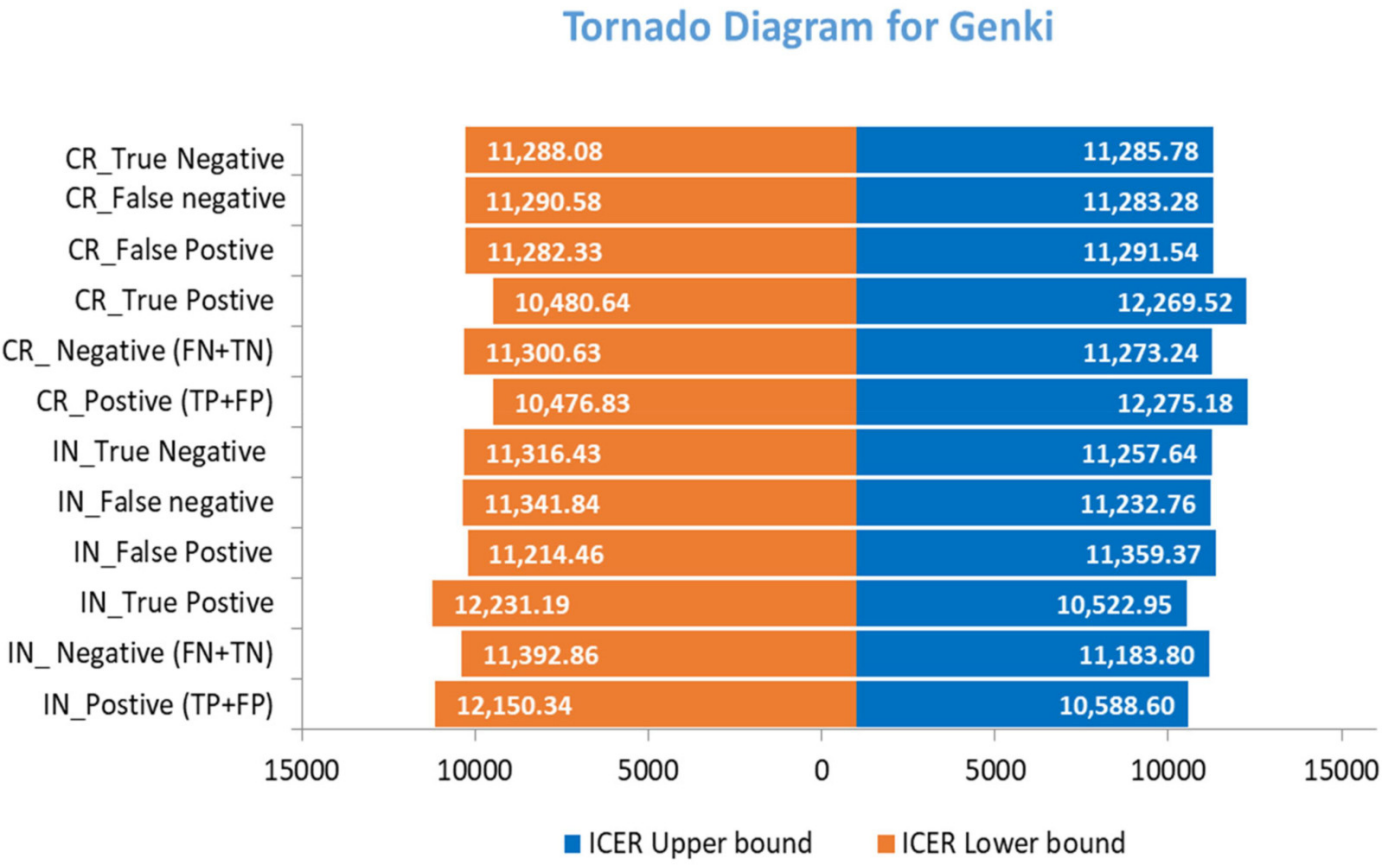
One-way sensitivity analysis for Genki.

The tornado diagram visually highlights the parameters that had the most significant impact on the ICER, aiding in identifying the key contributors to uncertainty. Minor changes were observed in multiple indicators when the ICER values were changed. In contrast, the cost and case outcomes were found to have a major influence on the model. However, the sensitivity analysis provided valuable insights at an acceptable level for decision-makers, guiding efforts to improve parameter estimation and reduce uncertainty in the cost-effectiveness analysis (Figures 5, 6).

### Threshold analysis

**Figure 7:**
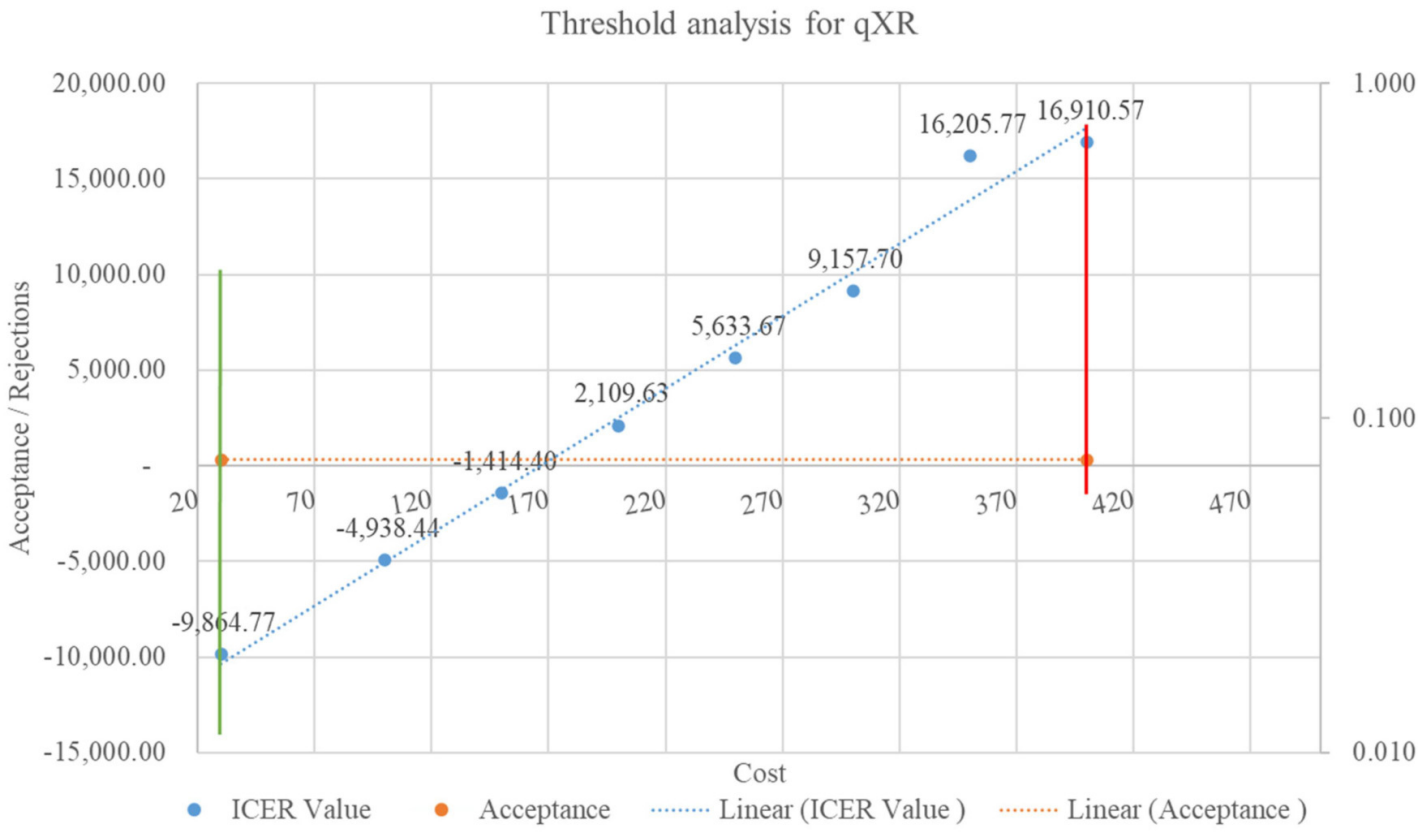
Threshold analysis for qXR.

**Figure 8:**
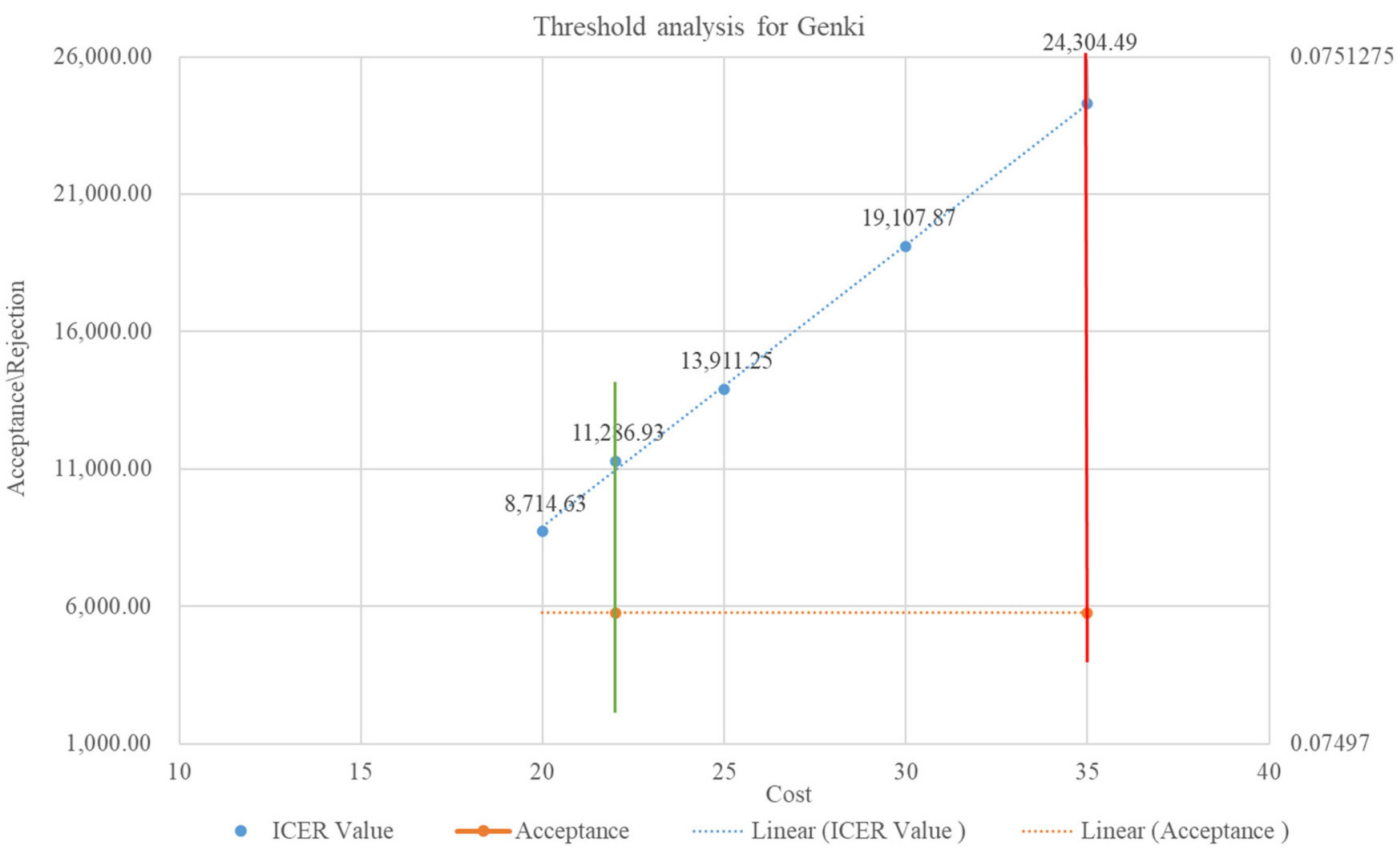
Threshold analysis for Genki.

The cost of the AI solution played a significant role in the overall expenses related to implementing AI screening tools in the program. The ICER values indicated that the AI solution qXR lowered costs when the price ranged from INR 30 (USD 0.36) to INR 410 (USD 5). In addition, the AI solution Genki remained cost-effective and acceptable when the price was INR 22 (USD 0.26). Both AI solutions are financially viable and provide value for money within the specified price ranges. However, when the cost per screening increased, the ICER values became positive, indicating a higher cost to obtain additional effectiveness. The healthcare system could invest a maximum of INR 35 (USD 0.42) for Genki and INR 410 (USD 5) for qXR to achieve one unit of health benefit. Figures 7 and 8 show the threshold analyses for qXR and Genki, respectively.

## Discussion

AI-assisted CXR interpretation can increase the accuracy and efficiency of TB diagnosis, as demonstrated by numerous studies (7). As per our study findings, both qXR and Genki demonstrated high pooled sensitivity and specificity compared to interpretation by a radiologist. The overall pooled sensitivity and specificity for the interventions were 90% and 68%, respectively. In other words, the AI-assisted technologies correctly identified 90% of TB cases, missed 10%, and correctly identified 68% of non-TB cases while falsely detecting TB in 32%. This pooled diagnostic accuracy met the non-inferior accuracy as per the WHO’s consolidated guidelines on systematic screening for tuberculosis (23).

The cost-effectiveness plane showed that both interventions fell within the acceptable quadrants Q1 (cost-saving and more effective) and Q2 (more effective but more costly). The high sensitivity and specificity of AI tools such as qXR and Genki, combined with their cost-effectiveness, make them a promising alternative to traditional radiologist interpretation. Given the cost-effectiveness and high diagnostic accuracy of qXR and Genki, there is significant potential for their wider adoption in TB screening programs.

Although radiologist interpretation is considered the gold standard for CXR interpretation; however, there is a shortage of radiologists relative to population size( i.e., 1:100,000), which poses a significant public health challenge (5). Radiologists are heavily overburdened, especially in public sector healthcare facilities, which may increase the risk of errors in reporting. Given these constraints, AI-assisted tools offer a promising solution to augment diagnostic capacity. Other studies have supported the integration of AI into radiology workflows, which optimizes the interpretation process and allows healthcare professionals to undertake triage of TB cases cost-effectively (24, 25).

The integration of AI tools into handheld, portable X-ray devices may be a breakthrough combination in TB diagnosis (26). Deploying this combination of AI tools and handheld, portable X-ray devices at the grassroots level, where a radiologist's presence cannot be expected, will enhance TB diagnosis.

This development will benefit underprivileged individuals who lack resources or face financial constraints and are unable to travel to community health centers or district hospitals, allowing them to receive timely TB screening, which ultimately reduces community transmission and leads to better health outcomes. A major concern of healthcare systems across the globe is rising out-of-pocket expenditures. This can be significantly reduced by leveraging AI in disease screening, diagnosis, or other aspects (27).

This study’s findings align with those of previous cost-effectiveness studies on AI-based solutions in other medical imaging applications. AI has demonstrated cost-effectiveness in CT screening for cardiovascular disease, osteoporosis, sarcopenia, diabetic retinopathy, early gastric cancers, and asymptomatic left ventricular dysfunction (28–31). These diverse applications highlight AI's potential to address resource constraints across multiple disease screening programs.

However, this study encountered a few limitations during data collation and analysis. This study was conducted from the provider's perspective and did not include matched case comparisons in the cost-effectiveness assessments. In addition, cost and programmatic data were obtained from the manufacturers and secondary sources, and the sampled population information was obtained from the user department.

## Conclusion

This rapid health technology assessment demonstrated that AI-assisted chest x-ray interpretation tools, qXR and Genki, are cost-effective alternatives to traditional radiologist interpretation for TB screening in India. These findings have important implications for India's National Tuberculosis Elimination Program. Incorporating AI tools into the interpretation of chest X-rays can increase the rate of early TB diagnosis, which will lead to timely treatment and better treatment outcomes. The implementation of these tools offers a promising alternative to traditional radiologist interpretation, particularly in resource-constrained settings such as India. Future primary studies focusing on comparing AI-based and gold standard interpretations would be useful.

## Data Availability

The data analyzed in this study are subject to the following licenses/restrictions: the data were provided by routine care sites and thus cannot be uploaded with the research article. Requests to access these datasets should be directed to Dhaval Parmar at dvparmar@gmail.com.
